# Role of micronucleus test in predicting breast cancer susceptibility: a systematic review and meta-analysis

**DOI:** 10.1038/bjc.2011.567

**Published:** 2011-12-20

**Authors:** F Cardinale, P Bruzzi, C Bolognesi

**Affiliations:** 1 Clinical Epidemiology Unit, National Cancer Research Institute, Largo R Benzi 10, 16132 Genoa, Italy; 2 Environmental Carcinogenesis Unit, National Cancer Research Institute, Largo R Benzi 10, 16132 Genoa, Italy

**Keywords:** micronucleus test, breast cancer, family history, BRCA1/2, systematic review

## Abstract

**Background:**

The cytokinesis-block micronucleus test (MNT), as a marker of chromosomal mutagen sensitivity, was applied in a number of studies enrolling breast cancer (BC) patients and subjects with known or putative genetic predisposition to BC. The large majority of them involve the evaluation of induced micronuclei (MN) frequency in peripheral lymphocytes, after the *in vitro* challenge with ionising radiations.

**Methods:**

The aim of the present systematic review and meta-analysis is to investigate the role of MN assay in the identification of individuals at increased risk of BC and its potential use as prescreening test in women with a family history (FH) of BC.

**Results:**

Twelve studies were included in the meta-analysis, covering a time interval 1998–2007, and including 752 cases and 593 controls. Among the cases, 629 are cancer patients and 123 are cancer-free subjects, including 32 first-degree relatives of the susceptible subjects and 91 BRCA1/2 mutation carriers. Our meta-analysis reveals a significant increase of baseline MN frequency related to cancer status, but the association with FH of BC and specifically with BRCA mutations is not clear. A larger difference in MN frequency between cases and controls was observed after *in vitro* challenge, but response to radiation exposure doesn't appear to better discriminate cancer-susceptible subjects.

**Conclusion:**

Our study suggests the presence of some bias affecting many of these studies, reinforcing the suggestion that a more rigorous study design is needed in this area.

The micronucleus test (MNT), as a marker of chromosomal damage, is a well-established assay in genetic toxicology and in human biomonitoring (Fenech, 2007). The MNT is one of the most widely applied assays to test new compounds, *in vitro* and *in vivo*, for regulatory purposes (OECD TG 474, 1997; OECD TG 487, 2009). The test is also successfully used in monitoring human populations exposed to genotoxic compounds; recommendations for the appropriate application are available (Albertini *et al*, 2000; Battershill *et al*, 2008). Recent prospective studies evaluating large cohorts of disease-free subjects revealed that an increase in micronuclei (MN) frequency in peripheral lymphocytes was associated with an increased risk of cancer, at the population level, providing a suggestive evidence that this biomarker could be predictive of cancer risk (Bonassi *et al*, 2007; Murgia *et al*, 2008).

Many papers were published on the application of the MN in peripheral lymphocytes of patients with cancer or preneoplastic lesions; the large majority of them show a significant increase of MN frequency in patients compared with control groups, with a large variability among the tumours and studies (Iarmarcovai *et al*, 2008; Bonassi *et al*, 2011; El-Zein *et al*, 2011). Increased MN frequency was also detected in peripheral lymphocytes of subjects affected by cancer-associated congenital syndromes characterised by deficiency in DNA damage response, such as Fanconi anaemia (Maluf and Erdtmann, 2001), Bloom syndrome or ataxia telangiectasia (Scott *et al*, 1996). Furthermore, the MN frequency in human peripheral lymphocytes or other surrogate tissues was shown to be influenced by genetic polymorphisms in various genes involved in DNA repair pathways or in xenobiotic metabolism, which are responsible for the interindividual differences in response to genotoxins (Dhillon *et al*, 2011). On the basis of these findings, the MN expression appears to be greatly modulated by the inherited and acquired deficiencies in host defence mechanisms against genotoxic compounds. Mutagen sensitivity, measured by quantifying the genotoxic events induced by chemical or physical agents in short-term cultures of peripheral blood lymphocytes, was used with the aim to improve the detection of the individual genetic susceptibility, reducing the influence of environmental factors. The heritability of mutagen sensitivity was shown in a number of studies involving first-degree relatives of cancer patients (Scott *et al*, 1998; Burrill *et al*, 2000) and in a classic twin study (Surowy *et al*, 2011).

A large proportion of the available studies on MN application as mutagen sensitivity assay was conducted in breast cancer (BC) patients and subjects with known or putative genetic predisposition to BC. Ionising radiation was the most frequently applied challenge agent in these studies, due to its potential to induce highly mutagenic genetic events allowing to estimate the inherited deficiency in DNA repair machinery, associated with known BC susceptibility genes.

Enhanced mutagen sensitivity, evaluated as the increase in MN frequency in peripheral lymphocytes from BC patients and subjects with a strong family history (FH) of BC, was observed in the initial studies (Scott *et al*, 1998, 1999; Burrill *et al*, 2000) and encouraged further investigations. Increase in MN radiation induced in small groups of healthy women carrying a BRCA1/2 mutation compared with matched control groups (Rothfus *et al*, 2000; Trenz *et al*, 2002, 2003) suggested a close relationship between the presence of these mutations and the MN induction by ionising radiations. Further studies didn't confirm this finding and showed an increased MN frequency associated to *in vitro* radiation exposure in sporadic BC patients (Varga *et al*, 2006), suggesting a different role of the assay.

A number of studies are now available on mutagen sensitivity, evaluated as an increased MN frequency in challenge assay, in different groups of sporadic and familial BC patients and in their relatives. Published studies are generally based on small size samples. As a consequence, comprehensive and reliable conclusions cannot be afforded by the results of individual studies, due to inadequate statistical power.

The aim of the present study was to retrieve, review and synthesise published evidence on this subject to define the role of this biomarker in the identification of individuals at increased risk of BC and its potential use as a prescreening test in women with a FH of BC.

## Materials and methods

This systematic review follows the methodology described in the PRISMA statement (Moher *et al*, 2009)

### Eligibility criteria

Eligible for the inclusion in the present review were all the case–control studies in which MNT was performed at baseline and after irradiation in women with BC or at risk of BC because of FH.

### Search strategy

Studies on the application of the MNT in BC patients with hereditary/familial factors were identified by using the MedLine/PubMed database (National Library of Medicine, National Institutes of Health, Bethesda, MD, USA; http://www.ncbi.nlm.nih.gov/pubmed/).

The terms ‘micronucleus’ and ‘micronuclei’ were used as medical subject heading associated to ‘breast neoplasm’, using AND operator. Only the studies including the radiation sensitivity assay were considered.

### Study selection

All retrieved studies were reviewed and assessed by two of us (CB and FC) for inclusion in the present analysis. A number of publications were discarded, because the groups of patients considered were partially or completely overlapping with those included in other studies from the same research groups. Only studies reporting an adequate experimental protocol with reference to established criteria of MN scoring were included in the meta-analysis.

### Data collection process

For each study, the following information was collected: the number of cases and respective controls, the MN frequency before and after irradiation with its s.e. where available, the clinical characteristics of the cases, the FH and the BRCA status, when assessed, and the different type and doses of irradiation.

### Classification of individual studies

Most of the studies clearly indicated the controls as negative or positive for all the three factors under study (tumour–FH–BRCA status). In the remaining studies, controls were arbitrarily considered negative for all the three factors, if no mention of positivity was made for any of them.

Other potential sources of heterogeneity are represented by the following: age and sex of subjects tested; method of subject recruitment; smoking habits; prior exposure to radiotherapy–chemotherapy; stage of the disease (early advanced); collection method of blood sample (timing, conservation and treatment); the experimental protocol; number of cells scored; type of irradiation (source and dose rate). All these information, when available, were recorded and discussed in the results section.

### Statistical analyses

The primary aim of this study was to assess the potential association between MN frequency, both at baseline and after irradiation, and presence of BC, FH of BC or a pathogenic mutation in a *BRCA* gene. Accordingly, within each study, the number of cases had to be identified that was relevant for each of these study questions. This led to the creation of subgroups of cases within each study. For each subgroup, cases were compared with the respective controls (subjects without BC, or with a negative FH for BC or negative/not evaluated for BRCA mutations).

From each publication, the MN frequency in each group of cases and controls, at baseline and after irradiation was abstracted. The primary contrasts examined were: (1) presence or absence of cancer (stratifying for FH); (2) positive or negative FH (stratifying for tumour status); (3) positivity or negativity/not evaluated for BRCA (stratifying for tumour status in FH-positive subjects).

From each study, the difference, with its s.e. and 95% confidence intervals (CI) at baseline and after irradiation, between cases and controls was computed. Log transformations, possibly more appropriate with MN frequency data, were not possible because individual data were not available.

Study-specific differences in MN frequency were pooled across studies; first within subgroups homogeneous for primary contrast and stratifying factors, and then across strata homogeneous for primary contrast by computing the weighted mean difference (WMD) with its 95% CI and *P*-value. The weight assigned to each study was 1/variance of the study-specific mean difference (Sutton *et al*, 2004). Meta-analysis was performed using a random effects model. Student's *t*-test was also performed to assess the significance of differences between means within subgroups. The heterogeneity within each set of values was examined with a standard *Q*-test statistic (testing the hypothesis of homogeneity) The results of these analyses are presented in three pairs of Forrest plots, each for a primary contrast, at baseline and after *in vitro* irradiation, where also stratum-specific, summary weighted differences are reported.

## Results

### Results of the literature search

**Figure 1:**
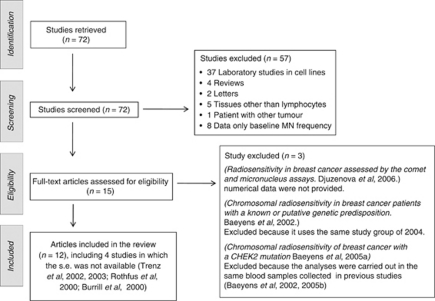
Flow chart: selection of the literature.

The literature search was carried out on March 2010. Its results and the stepwise exclusion process is described in Figure 1. A total of 75 papers were retrieved and screened; 57 of them were discarded because they were not pertinent to our analysis for a number of reasons, as described in the flow chart.

Fifteen full-text articles were assessed for eligibility and three of them were excluded because the numerical results were not provided (Djuzenova *et al*, 2006), or because the study groups had been already used in previous studies (Baeyens *et al*, 2002, 2005b) As a consequence, 12 articles (Table 1) were left for the inclusion in the review, including 4 studies in which the s.e. was not available (Burrill *et al*, 2000; Rothfus *et al*, 2000; Trenz *et al*, 2002, 2003).

### Description of the studies

The characteristics of the selected studies are reported in Table 1. A total of 752 BC cases and 593 controls were included in the analysis. A total of 629 cases are BC patients; 266 of them were reported as unselected and 274 as sporadic, 11 as index cases; 78 are cases with FH, 26 as BRCA1/2 positive, 52 as BRCA1/2 negative. A total of 123 cases were cancer-free; it included 22 first-degree relatives of the index cases, 57 women with mutation (BRCA1/2), 34 relatives with mutation (BRCA1/2) and 10 relatives without mutation. A total of 593 controls were reported as healthy subjects/normal volunteers, 44 were defined as subjects without any FH of cancer and 25 as mutation-negative females from the same families of the cases. Age of cases and controls including the respective ranges is reported only in nine studies.

The blood samples were collected at different times before, during or after the chemotherapy/radiotherapy. For 396 BC patients, no exposure to chemotherapy/radiotherapy during or before sampling was reported; 69 were exposed to radiotherapy before or during sampling; one study reported that 47 cases were sampled between diagnosis and start of the therapy or when therapy was finished.

Only two studies collected data on health status, lifestyle factors and presence of concomitant therapies (Trenz *et al*, 2002; Kotsopoulos *et al*, 2007). In seven studies, the previous or concomitant use of antibiotics, tamoxifen or radio-chemotherapy was considered (Scott *et al*, 1998, 1999; Baeyens *et al*, 2004, 2005b; Ban *et al*, 2004; Mozdarani *et al*, 2005; Varga *et al*, 2006). Smoking habits were collected in only three studies (Ban *et al*, 2004; Varga *et al*, 2006; Kotsopoulos *et al*, 2007).

In different subgroups, the frequency of MN in cases and controls was analysed only at baseline or only after irradiation (at different dose rate) or in both cases.

### Laboratory methods

All the studies selected for the meta-analysis applied the cytokinesis-block micronucleus assay (Fenech, 2007). The experimental protocols used were very heterogeneous (Table 1). High doses (1–3.5 Gy) of ionising *x*- or γ-radiations from different sources, cobalt 60 or caesium 137, were used as challenge agents for the *in-vitro* sensitivity assay.

Different irradiation procedures were adopted in the different studies: low-dose rate (0.10–0.40 cGy min^−1^) or high-dose rate (0.7–4 Gy min^−1^).

Different times between the irradiation and cell culture (0–3 h) and different harvesting times (from 69–90 h) were applied in the studies. All the studies refer to established scoring criteria, although none considered the most advanced cytome protocol recently proposed (Fenech, 2007). All the studies reported the scoring of 1000 binucleated cells for MN analysis, with the exception of Scott *et al* (1998, 1999), Burrill *et al* (2000) and Kotsopoulos *et al* (2007), where 100–200 cells were analysed. No statistical significant difference in mean MN frequency (Student's *t*-test) was observed in studies scoring 100–200 cells. We didn't consider this deviation from the established MN protocol as a prejudice for the inclusion in our meta-analysis.

One study reports also an exercise on a subgroup of subjects using the automatic system for MN scoring.

### Subgroups of patients and controls

A total of 51 different subgroups of cases, defined by their characteristics (presence of BC, presence of a FH of BC and presence of a pathogenic mutation in a *BRCA* gene) were identified within the 12 studies (Table 2). For each subgroup, cases were compared with the controls (subjects without BC, and with a negative FH for BC and negative/not evaluated for BRCA mutations).

Student's *t-*test was performed to evaluate differences between high-dose rate and low-dose rate subgroups. The test did not show statistically significant differences between the two groups.

Funnel plots of all studies, at baseline and on irradiated cells, failed to provide any evidence of publication bias, even though they confirmed the large variability of results across studies.

### MN frequency in different subgroups

**Figure 2:**
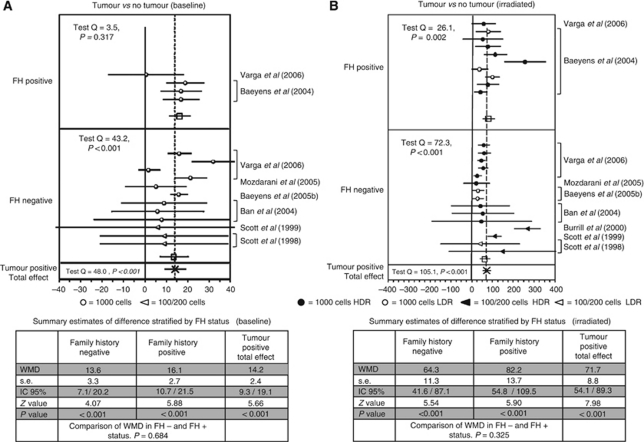
(**A** and **B**) Forest plot of the differences in the baseline and induced MN frequency between subjects with breast cancer and healthy controls. Studies are plotted in order of publication. Each circle represents the difference between cases and controls. Horizontal lines corresponds to 95% CI. The square symbolises the WMD (weighted mean difference) for each subgroup.

*Presence* vs *absence of BC* There were 16 contrasts available to assess the difference in the baseline MN frequency between subjects with BC and healthy controls (Figure 2A). Twelve contrasts were in FH-negative and four in FH-positive cases. In all studies, MN frequency was higher in cases than in controls. Overall, there was a difference of 14.2 MN/1000 (s.e. 2.4; 95% CL 9.3–19.1; *P*<0.001) between BC patients and controls (subjects without cancer, BRCA negatives and with negative FH for BC).

The difference was similar in FH-negative subjects (WMD 13.6; s.e. 3.3; 95% CL 7.1–20.2; *P*<0.001) and in FH-positive subjects (WMD 16.1; s.e. 2.7; 95% CL 10.7–21.5; *P*<0.001). The difference between means of the two subgroups was not significant (Student's *t*-test *P*=0.684), suggesting that the increase in MN frequency was due solely to the presence of cancer (Figure 2A).

The difference between BC cases and controls in MN frequency after irradiation of the cell culture was evaluated in 25 contrasts; 15 in FH-negative subjects and 10 in FH-positive subjects (Figure 2B). Again, MN frequency was higher in BC cases than in controls in all contrasts, for a summary WMD of 71.7 (s.e.=8.8, 95% CL 54.1–89.3; *P*<0.001). The increase observed in FH-positive subjects (WMD 82.2; s.e. 13.7; 95% CL 54.8–109.58; *P*<0.001) compared with that observed in FH-negative subjects (WMD 64.3; s.e.=11.3; 95% CL 41.6–87.1; *P*<0.001) could suggest a possible effect of hereditary/familial factors, but the difference between the two WMD's groups was not significant (Student's *t*-test *P*=0.325).

**Figure 3:**
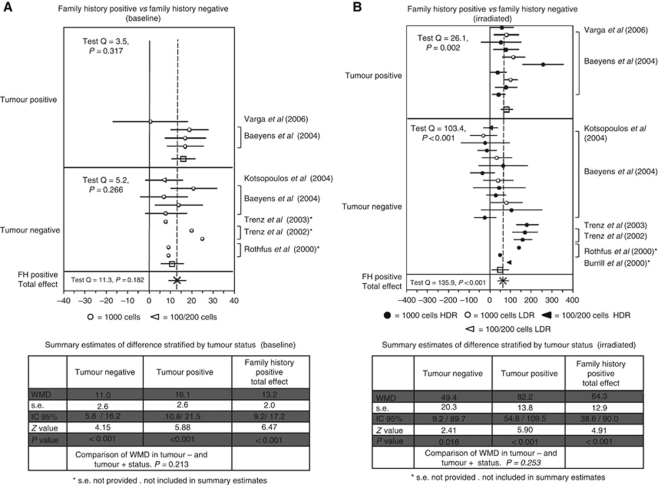
(**A** and **B**) Forest plot of the differences in the baseline and induced MN frequency between subjects with a FH for BC and healthy controls. Studies are plotted in order of publication. Each circle represents the difference between cases and controls. Horizontal lines corresponds to 95% CI. The square symbolises the WMD (weighted mean difference) for each subgroup.

*FH positive* vs *negative* There were 14 contrasts available to assess the difference in the baseline MN frequency between subjects with positive and negative FH (Figure 3A). Ten contrasts were in tumour-negative subjects (with five contrasts excluded because of missing information on s.e.) and four in tumour-positive subjects. In all studies, MN frequency was higher in cases than in controls. Overall, there was a difference of 13.2 MN/1000 (s.e. 2.0; 95% CL 9.2–17.2; *P*<0.001) between positive FH subjects and controls.

At baseline, the difference between FH positive and FH negative was higher in tumour-positive subjects (WMD 16.1; s.e. 2.6; 95% CL 10.8–21.5; *P*<0.001) than in tumour-negative subjects (WMD 11.0; s.e. 2.6; 95% CL 5.8–16.2; *P*<0.001). The increment in MN frequency in the tumour-positive subjects could suggest an independent effect of the two factors, (Figure 3A) although the Student's *t*-test (*P*=0.213) is not significant.

The difference in MN frequency after irradiation of the cell culture between subjects with positive and negative FH was evaluated in 29 contrasts; 19 in tumour-negative subjects (with 3 contrasts excluded because of missing information on s.e.) and 10 in tumour-positive subjects (Figure 3B). MN frequency was higher in subjects with positive FH than in controls in 24 contrasts and lower in 5, for a summary WMD of 64.3 (s.e.=12.9; 95% CL 38.6–90.0; *P*<0.001). The increase observed in tumour-positive subjects (WMD 82.2; s.e. 13.8; 95% CL 54.8–109.5; *P*<0.001) compared with that observed in tumour-negative subjects (WMD 49.4; s.e. 20.3; 95% CL 9.2–89.7; *P*=0.016), could suggest a more marked effect of the cancer patient status, but the difference between the two WMD's was not significant (Student's *t*-test *P*=0.253).

**Figure 4:**
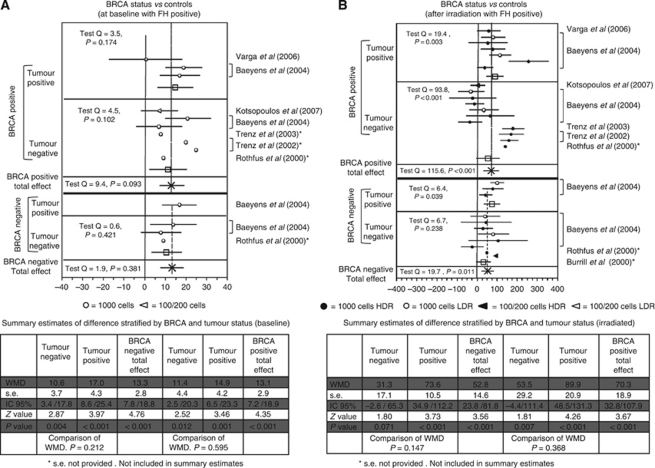
(**A** and **B**) Forest plot of the differences in the baseline and induced MN frequency between BRCA1/2 mutation carriers and healthy controls. Studies are plotted in order of publication. Each circle represents the difference between cases and controls. Horizontal lines corresponds to 95% CI. The square symbolises the WMD (weighted mean difference) for each subgroup.

*Role of BRCA* There were 14 contrasts available to assess the difference in the baseline MN frequency between subjects with positive FH in whom BRCA status was assessed and healthy controls (Figure 4A).

Three contrasts were in BRCA negative – tumour-negative subjects (with one contrast excluded because of missing information on s.e.); one contrast in BRCA negative – tumour-positive subjects; seven in BRCA positive – tumour-negative subjects (with four contrasts excluded because of missing information on s.e.) and three contrasts in BRCA positive – tumour-positive subjects.

In all studies, MN frequency was higher in cases than in controls.

Overall, in the BRCA-negative group, there was a difference of 13.3 MN/1000 (s.e. 2.8; 95% CL 7.8–18.8, *P*<0.001) between BRCA-negative patients with FH positive and controls. In the BRCA-positive group, the difference was almost identical; 13.1 MN/1000 (s.e. 2.9; 95% CL 7.2–18.9, *P*<0.001) between BRCA-positive patients with FH positive and controls (subjects without cancer, BRCA negative and with a negative FH for BC).

At baseline, when the effect of BRCA status on MN frequency was stratified according to BC status, no noteworthy variations was seen; in fact, the difference was similar in BRCA negative – tumour-negative subjects (WMD 10.6; s.e. 3.7; 95% CL 3.4–17.8; *P*=0.004) and in BRCA positive – tumour-negative subjects (WMD 11.4; s.e. 4.4; 95% CL 2.5–20.3; *P*=0.012).

Also in subjects with BC, the difference between cases and controls was similar in BRCA-positive (WMD 14.9; s.e. 4.2; 95% CL6.5–23.3; *P*=0.001) and BRCA-negative subjects (WMD 17.0; s.e. 4.3; 95% CL 8.6–25.4; *P*<0.001).

There were 28 contrasts available to assess the difference in the MN frequency, after irradiation of the cell culture, between subjects with positive FH, in whom BRCA status had been assessed, and healthy controls. Seven contrasts were in BRCA negative – tumour-negative subjects (with 1 contrast excluded because of missing information on s.e.); 3 in BRCA negative – tumour-positive subjects; 11 in BRCA positive – tumour-negative subjects (with 1 contrast excluded because of missing information on s.e.) and 7 in BRCA positive – tumour-positive subjects (Figure 4B).

In all contrasts but five, MN frequency was higher in cases than in controls. Overall, in the BRCA-negative group, there was a difference of 52.8 MN/1000 (s.e. 14.6; 95% CL 23.8–81.8; *P*<0.001) between negative BRCA patients with FH positive and controls (subjects without cancer, BRCA negatives and with negative FH for BC).

In the entire BRCA-positive group, there was a similar difference of 70.3 MN/1000 (s.e. 18.9; 95% CL 32.8–107.9, *P*<0.001) between positive BRCA patients with FH positive and controls (subjects without cancer, BRCA negatives and with negative FH for BC).

The role of BRCA status was then evaluated after stratification for the presence/absence of BC. In BC patients, the difference between cases and controls was very similar in BRCA-negative subjects (WMD 73.6; s.e. 10.5; 95% CL 34.9–112.2; *P*<0.001) and in BRCA-positive subjects (WMD 89.9; s.e. 20.9; 95% CL 48.5–131.3; *P*<0.001). There is no significant difference between the WMD of the two groups (Student's *t-*test *P*=0.641).

Conversely the increase observed in BRCA positive – tumour-negative subjects (WMD 53.5; s.e. 29.2; 95% CL −4.4–111.4; *P*=0.007) was almost twice as large as that in BRCA negative – tumour-negative subjects (WMD 31.3; s.e. 17.1; 95% CL −2.6–65.3; *P*=0.071). This could suggest an effect of BRCA on MN frequency, but again, the difference between the two WMD was not significant (Student's *t-*test *P*=0.590).

## Discussion

The MNT has been widely applied to evaluate the chromosomal instability in selected groups of patients affected by cancer or degenerative diseases, compared with control, healthy populations (Andreassi *et al*, 2011; Bonassi *et al*, 2011; El-Zein *et al*, 2011; Migliore *et al*, 2011). Furthermore, the induced MN frequency, as expression of mutagen sensitivity, was viewed as a possible means to predict the BC risk at the individual level in the light of the link between deficient repair of DNA and genetic predisposition to BC. Known BC susceptibility genes such as *BRCA1, BRCA2, ATM, CHK2* and *TP53* are involved in maintaining the genomic stability through their involvement in DNA double-strand break repair, transcription-coupled repair and homologous recombination; defects in their expression are expected to lead to an increased mutagen sensitivity as expression of an intermediate phenotype in cancer development (Venkitaraman, 2002, 2009; Smith *et al*, 2003; Hsu *et al*, 2007; Ralhan *et al*, 2007; Couch and Wang, 2009; Kwei *et al*, 2010; Latimer *et al*, 2010).

Many studies are available in the literature on the application of MNT, evaluated as baseline level (Iarmarcovai *et al*, 2008; Bonassi *et al*, 2011) and after *in vitro* challenge, involving more than 1000 cases of BC patients and subjects with known or putative cancer predisposition. An association between MN induction and BC development was reported in the large majority of studies showing an increased baseline MN frequency in untreated cancer patients. Enhanced *in-vitro* mutagen sensitivity, revealed by the MN assay, in BC patients compared with healthy controls is also a common finding with large variability among studies, whereas contrasting results were obtained in subjects with FH. Few studies involving small groups of subjects show increased mutagen sensitivity in irradiated peripheral lymphocytes of BRCA1/2 mutation carriers compared with healthy controls, but the implication of the BRCA mutation was not appreciable in other studies when the comparison was made with BC patients without a BRCA mutation, suggesting a major role of cancer status in mutagen sensitivity.

Overall, despite numerous studies showing the enhanced chromosomal damage as a cancer predisposition factor, the role of MNT in predicting BC risk is still not clear.

The main reasons for that are the inadequacy of the study design, the inconsistency in subject recruitment and classification, and the small size of most studies.

Our systematic review, focusing on investigations involving the *in vitro* challenge, selected 12 studies eligible for the meta-analysis. These studies, published between 1998 and 2007, are very heterogeneous in many respects. First, tumour status, FH and BRCA status were not homogenously characterised. Cancer patients were categorised as unselected or sporadic. The criteria applied to define the FH were different, including in some cases an early onset of cancer and in one case, previous data on radiation sensitivity. BRCA-positive cases include cancer patients and their relatives carrying different BRCA1 or BRCA2 mutations.

A second factor that was not consistently considered in the different studies is the exposure to genotoxic agents, antiblastic drugs or radiations, for therapy or diagnostic purposes. The chromosomal damage associated to these treatments could last long time after the exposure, affecting the baseline MN frequency and potentially modulating the responses to the challenge agent *in vitro*. The data available on patients who received a cytostatic therapy show that persistent DNA damage in lymphocytes before culture can be expressed as MN during the *in-vitro* cell proliferation. A large variability in the persistence of chromosomal damage after a chemotherapeutic treatment was described, related to the kind of drug, the period of treatment and the individual response (Arsoy *et al*, 2009).

Finally, the experimental protocols applied in the selected studies were heterogeneous; different criteria of MN scoring were used, the conditions of *in-vitro* irradiation included different doses and low- and high-irradiation rate. All these factors increase the variability within and among the studies.

Despite all these factors potentially impairing this meta-analysis, our review indicates that the chromosomal mutagen sensitivity, measured as MN frequency, is a common feature of cells from BC patients, but it is not clearly associated with FH and specifically with BRCA mutations. A significantly increased baseline MN frequency was observed in more than 700 BC patients pooled from the different studies, when compared with healthy controls. Conversely, the role of the genetic predisposition to BC could not be demonstrated in a smaller and more heterogeneous pool of 201 subjects characterised by a cancer FH, including cancer patients and healthy subjects. Higher difference in MN frequency was observed in the subgroup of cancer patients than in healthy subjects, confirming cancer status as the main determinant. The available studies on BRCA 1 and 2 mutation carriers involved small groups of subjects and reported contrasting results; our meta-analysis including 117 cases, pooled from different studies, shows similar difference between cases and controls in BRCA-positive cases and in cases with a negative BRCA test.

A large difference between cases and controls was detected in all subgroups, using the challenge assay. However, this approach did not appear to improve the possibility to identify cancer-susceptible subjects on the basis of the response to radiation exposure; the differences between cases and controls was not significantly increased in family positive and BRCA positive as compared with family negative and BRCA negative.

The results of our analysis, far from being conclusive, suggest the need of further well-designed studies to define the role of MNT and the advantage of using *in-vitro* challenge in predicting individual BC risk. Of the various factors highlighted by our meta-analysis, as relevant in designing new studies, the most important ones are the procedures for the recruitment of study subjects and the characterisation of cases and controls. Other factors contributing to the difference in test performance among the studies are related to the use of different experimental protocols and criteria of scoring. The use of a standardised protocol and the intercalibration of the MN scoring among the labs in multicentric studies, needed to recruit adequate groups of cases, could allow to reduce the test variability. In addition, the perspective to apply the automated scoring of MN (Varga *et al*, 2004; Decodier *et al*, 2009; Willems *et al*, 2010; Bolognesi *et al*, 2011) could allow to evaluate large number of cultures and cells allowing to minimise the experimental variability and to define more reliable individual MN frequency values.

In summary, the systematic difference in MN frequency between cases and controls, in the large majority of contrasts, not attributable to any of the factors under study, suggests the presence of some bias affecting many of these studies, thus reinforcing the suggestion that more rigorous study designs are needed in this area.

## Figures and Tables

**Table 1:** Characteristics of the 12 studies included in the meta-analysis on MN and breast cancer

**Reference**	**Contrast N in Table 2**	**Cases**	**Controls**	**MN assay**	**Treatment**
Scott *et al* (1998)	1–2	39 breast cancer patients Without any exposure to chemotherapy or radiotherapy before sampling Age 58.5 (7.4) range (35–70)	42 normal volunteers (28 women and 14 men) Age 47.8 (13.4) range (23–72)	Whole blood PHA 6 h after irradiation Harvesting 90 h Slides stained with Giemsa Visual counting 100 BNCs/slide	Gamma rays source: 137 Cs total dose 3.5 Gy HDR dose rate 1 Gy min^−1^ LDR dose rate 0.15 cGy min^−1^
					
Scott *et al* (1999) Extension of the previous study (Scott *et al*, 1998)	3	130 breast cancer patients Without any exposure to cytotoxic chemotherapy before sampling Age 57 range (30–75)	68 healthy subjects Age 45 range (22–72)	Whole blood PHA 6 h after irradiation Harvesting 90 h Slides stained with Giemsa Visual counting 100 BNCs/slide	Gamma rays source:137Cs total dose 3.5 Gy HDR dose rate 1 Gy min^−1^
					
Burrill *et al* (2000)	4–5	11 index cases (level of radiation sensitivity > cut off) The cases were selected among the sensitive cases from breast cancer patients (Scott *et al*, 1999) 22 first-degree relatives of the index cases	68 healthy subjects historical controls from Scott *et al* (1999)	Whole blood PHA 6 h after irradiation Harvesting 90 h Slides stained with Giemsa Visual counting 100 BNCs/slide	Gamma rays source: 137 Cs total dose 3.5 Gy HDR dose rate 1 Gy min^−1^
					
Rothfus *et al* (2000)	6–7	22 members of 13 families with a familial BRCA1 mutation Age 42 (10) range 23–58	17 age-matched women without any family history of cancer Age 36 (9) range 25–53	Whole blood Harvesting 68 h Slides stained with acridine orange Visual counting 1000 BNCs/slide	Gamma rays source: cobalt-60 total dose 2 Gy HDR dose rate 4 Gy min^−1^
					
Trenz *et al* (2002)	8–9	10 women carrying a familial BRCA1 mutation 9 women with a familial BRCA2 mutation.	14 women without any family history of cancer	Whole blood Harvesting 68 h Slides stained with acridine orange Visual counting 1000 BNCs/slide	Gamma rays source cobalt-60 total dose 2 Gy HDR dose rate4 Gy min^−1^
					
Trenz *et al* (2003)	10	13 women carrying a familial BRCA1 mutation	13 women without any family history of cancer	Whole blood Harvesting 68 h Slides stained with acridine orange Visual counting 1000 BNCs/slide	Gamma rays source cobalt-60 total dose 2 Gy HDR dose rate4 Gy min^−1^
					
Ban *et al* (2004)	11–13	130 unselected breast cancer patients 58 without radiotherapy 69 radiotherapy during and before blood sampling Age 53.2 (10.8) range (26–78)	48 healthy women Age 46 (9.8) range (23–60)	Whole blood Harvesting 70 h Slides stained with Giemsa Visual counting Total 1000 BNCs	*x* rays Total dose 2 Gy HDR dose rate 1 Gy min^−1^
					
Baeyens *et al* (2004)	14–41	Breast cancer patients: 52 with family history 11 BRCA1 9 BRCA2 Relatives with mutation 6 BRCA1 6 BRCA2 Relatives without mutation: 5 BRCA1 5 BRCA2 Age 45 (10) range (29–69)	57 healthy women Age 37(12) range (29–69)	Whole blood Harvesting 70 h Slides stained with Giemsa Visual counting 1000 BNCs /subject	Gamma rays source cobalt-60 total dose 2 Gy HDR1 Gy min^−1^ total dose 3.5 Gy HDR 1 Gy min^−1^ Total dose 3.5 Gy LDR; 4 mGy min^−1^
					
Baeyens *et al* (2005b)	42–43	86 unselected breast cancer patients, sampling was carried out before surgery and chemotherapy Age 51 (11) range (31–77)	84 normal healthy women 73 concurrent control Age 30 (7) range 24–58	Whole blood Harvesting 70 h Slides stained with Giemsa Visual counting 1000 BNCs /subject	Gamma rays source cobalt-60 total dose 3.5 Gy LDR; 4 mGy min^−1^
					
Mozdarani *et al* (2005)	44	50 unselected breast cancer patients (1 man and 49 women) Without any exposure to chemotherapy or radiotherapy before sampling Age 46.9 (11.4) range (25–78) All no smokers	40 normal healthy subjects (13 men and 27 women) Age 38.1 (9.4) range (24–62) All no smokers	Whole blood Harvesting 90 h Slides stained with Giemsa Visual counting 1000 BNCs/slide	Gamma rays source cobalt-60 total dose 3 Gy HDR dose rate 70 cGy min^−1^
					
Varga *et al* (2006)	45–50	Sample A: 85 sporadic breast cancer 6 carriers of BRCA1 mut 47 between diagnosis and start of the therapy or when therapy had been finished for at least 3 months 33 without therapy Age 56.7 range (35–80) 32 sporadic cancers (subgroup) Age 56.6 range (37–80) Sample B: 20 sporadic breast cancer patients Age 44.4 range (23–68)	Sample A: 96 female controls hospital healthy patients non-blood relatives of the patients and occasional controls Age 42.7 range (20–90) 21 controls (subgroup) age 40.5 range (23–68) Sample B: 21 hospital healthy patients (recruited in another hospital) Age 58.0 range (29–81)	Whole blood culture Harvesting 72 h Slides stained with Giemsa Visual counting 500–1000 BNCs Automated counting (DAPI) 500 BNCs; reanalysis Automated counting (DAPI) 500 BNCs	Gamma rays source: 137 CS total dose 2 Gy HDR dose rate 4 Gy min^−1^
					
Kotsopoulos *et al* (2007)	51	25 cancer-free female heterozygous BRCA1 mutation carriers Age 43.56 (9.81) range (20–60)	25 healthy mutation-negative females from the same families as the cases Age 44.62 (11.19) range (20–60)	Isolated lymphocytes Irradiated 24 h after the culture set up Harvesting 68 h Slides stained with acridine orange Visual counting 200 BNCs	Gamma rays source: 137 CS total dose 2 Gy HDR 1.07 Gy min^−1^

Abbreviations: BNCs=binucleated cells; DAPI=4',6-diamidino-2-phenylindole; HDR=high-dose rate; LDR=low-dose rate; MN=micronuclei; Neg=negative; PHA=phytohemoagglutinin.

**Table 2:** List of contrasts in individual studies

	**Author**	**Cases**	**Controls**	**Dose**	**Tumour status**	**Family history**	**BRCA status^a^**	**Dose rate^b^**	**N° cells^c^**	**Diff.B^d^**	**UCI 95% (B)^e^**	**LCI 95% (B)^f^**	**Weight (B)^g^**	**Diff.I^h^**	**UCI 95% (I)^i^**	**LCI 95% (I)^j^**	**Weight (I)^k^**
1	Scott *et al* (1998)	39	42	3.5	Yes	No	Neg	HDR	100	9.0	38.6	−20.6	0.0044	149.0	405.3	−107.3	0.0001
2		39	42	3.5	Yes	No	Neg	LDR	100	9.0	38.6	−20.6	0.0044	42.0	231.5	−147.5	0.0001
																	
3	Scott *et al* (1999)	130	68	3.5	Yes	No	Neg	HDR	100	6.0	66.3	−54.3	0.0011	110.0	141.5	78.5	0.0039
																	
4	Burrill *et al* (2000)	11	68	3.5	Yes	No	Neg	HDR	100	—	—	—	—	269.0	331.8	206.2	0.0010
5		22	68	3.5	No	Yes	Neg	HDR	100	—	—	—	—	92.0	—	—	—
																	
6	Rothfus *et al* (2000)	10	17	2	No	Yes	Neg	HDR	1000	9.3	—	—	—	51.0	—	—	—
7		12	17	2	No	Yes	Pos	HDR	1000	9.3	—	—	—	142.0	—	—	—
																	
8	Trenz *et al* (2002)	10	14	2	No	Yes	Pos	HDR	1000	25.0	—	—	—	160.0	202.8	117.2	0.0021
9		9	14	2	No	Yes	Pos	HDR	1000	20.0	—	—	—	170.0	229.9	110.1	0.0011
																	
10	Trenz *et al* (2003)	13	13	2	No	Yes	Pos	HDR	1000	8.0	—	—	—	180.0	232.7	127.3	0.0014
																	
11	Ban *et al* (2004)	130	48	2	Yes	No	Neg	HDR	500	8.0	40.0	−24.0	0.0037	49.0	289.4	−191.4	0.0001
12		58	48	2	Yes	No	Neg	HDR	500	6.0	27.7	−15.7	0.0081	55.0	202.8	−92.8	0.0002
13		69	48	2	Yes	No	Neg	HDR	500	9.0	29.4	−11.4	0.0092	43.0	181.8	−95.8	0.0002
																	
14	Baeyens *et al* (2004)	42	40	0	Yes	Yes	Neg	N	1000	17.0	25.4	8.6	0.0546	—	—	—	—
15		5	40	0	No	Yes	Neg	N	1000	8.0	17.6	−1.6	0.0415	—	—	—	—
16		5	40	0	No	Yes	Neg	N	1000	14.0	25.0	3.0	0.0318	—	—	—	—
17		11	40	0	Yes	Yes	Pos	N	1000	17.0	26.5	7.5	0.0430	—	—	—	—
18		4	40	0	No	Yes	Pos	N	1000	7.0	18.1	−4.1	0.0312	—	—	—	—
19		6	40	0	Yes	Yes	Pos	N	1000	19.0	27.6	10.4	0.0516	—	—	—	—
20		6	40	0	No	Yes	Pos	N	1000	21.0	31.8	10.2	0.0328	—	—	—	—
21		52	51	3.5	Yes	Yes	Neg	LDR	1000	—	—	—	—	101.0	133.4	68.6	0.0037
22		5	51	3.5	No	Yes	Neg	LDR	1000	—	—	—	—	81.0	156.6	5.4	0.0007
23		5	51	3.5	No	Yes	Neg	LDR	1000	—	—	—	—	41.0	112.1	−30.1	0.0008
24		10	51	3.5	Yes	Yes	Pos	LDR	1000	—	—	—	—	115.0	168.3	61.7	0.0014
25		5	51	3.5	No	Yes	Pos	LDR	1000	—	—	—	—	35.0	106.3	−36.3	0.0008
26		8	51	3.5	Yes	Yes	Pos	LDR	1000	—	—	—	—	81.0	140.3	21.7	0.0011
27		6	51	3.5	No	Yes	Pos	LDR	1000	—	—	—	—	−29.0	35.5	−93.5	0.0009
28		5	37	2	No	Yes	Pos	HDR	1000	—	—	—	—	−12.0	33.2	−57.2	0.0019
29		6	57	3.5	No	Yes	Pos	HDR	1000	—	—	—	—	−22.0	93.9	−137.9	0.0003
30		22	37	2	Yes	Yes	Neg	HDR	1000	—	—	—	—	43.0	74.1	11.9	0.0040
31		51	57	3.5	Yes	Yes	Neg	HDR	1000	—	—	—	—	79.0	130.1	27.9	0.0015
32		4	37	2	No	Yes	Neg	HDR	1000	—	—	—	—	−23.0	29.7	−75.7	0.0014
33		4	57	3.5	No	Yes	Neg	HDR	1000	—	—	—	—	105.0	250.6	−40.6	0.0002
34		5	37	2	No	Yes	Neg	HDR	1000	—	—	—	—	31.0	75.8	−13.8	0.0019
35		5	57	3.5	No	Yes	Neg	HDR	1000	—	—	—	—	45.0	170.3	−80.3	0.0002
36		10	37	2	Yes	Yes	Pos	HDR	1000	—	—	—	—	40.0	77.8	2.2	0.0027
37		9	57	3.5	Yes	Yes	Pos	HDR	1000	—	—	—	—	258.0	355.7	160.3	0.0004
38		3	37	2	No	Yes	Pos	HDR	1000	—	—	—	—	−34.0	23.6	−91.6	0.0012
39		6	57	3.5	No	Yes	Pos	HDR	1000	—	—	—	—	65.0	182.6	−52.6	0.0003
40		3	37	2	Yes	Yes	Pos	HDR	1000	—	—	—	—	79.0	139.1	18.9	0.0011
41		9	57	3.5	Yes	Yes	Pos	HDR	1000	—	—	—	—	55.0	150.7	−40.7	0.0004
																	
42	Baeyens *et al* (2005b)	73	73	3.5	Yes	No	Neg	LDR	1000	—	—	—	—	29.0	56.2	1.8	0.0052
43		86	84	3.5	Yes	No	Neg	LDR	1000	16.0	20.2	11.8	0.2213	32.0	58.2	5.8	0.0056
																	
44	Mozdarani *et al* (2005)	50	40	3	Yes	No	Neg	HDR	1000	5.3	19.4	−8.8	0.0193	24.0	85.6	−37.6	0.0010
																	
45	Varga *et al* (2006)	20	21	2	Yes	No	Neg	HDR	500	—	—	—	—	28.3	47.3	9.3	0.0107
46		32	21	2	Yes	No	Neg	HDR	500	1.9	6.9	−3.1	0.1559	47.9	66.1	29.7	0.0116
47		85	96	2	Yes	No	Neg	HDR	1000	21.4	28.7	14.1	0.0723	56.4	78.2	34.6	0.0081
48		6	96	2	Yes	Yes	Pos	HDR	1000	0.4	18.1	−17.3	0.0123	58.3	115.3	1.3	0.0012
49		47	96	2	Yes	No	Neg	HDR	1000	32.1	41.9	22.3	0.0397	61.7	91.4	32.0	0.0044
50		33	96	2	Yes	No	Neg	HDR	1000	16.3	24.7	7.9	0.0539	59.5	86.0	33.0	0.0055
																	
51	Kotsopoulos *et al* (2007)	25	25	2	No	Yes	Pos	HDR	200	7.1	15.8	−1.7	0.0498	3.9	37.8	−29.9	0.0034

Abbreviations: HDR=high-dose rate; LCI=lower confidence interval; LDR=low-dose rate; Neg=negative; Pos=positive; UCI=upper confidence interval.

a Negative if BRCA is negative for BRCA1 or 2, and positive if BRCA is positive for BRCA 1 or 2.

b HDR or LDR.

c Number cells analysed.

d Difference between cases and controls at baseline.

e UCI at baseline.

f LCI at baseline.

g Weight at baseline.

h Difference between cases and controls after irradiation.

i UCI after irradiation.

j LCI after irradiation.

k Weight after irradiation.
